# Confidence and psychosis: a neuro-computational account of contingency learning disruption by NMDA blockade

**DOI:** 10.1038/mp.2015.73

**Published:** 2015-06-09

**Authors:** F Vinckier, R Gaillard, S Palminteri, L Rigoux, A Salvador, A Fornito, R Adapa, M O Krebs, M Pessiglione, P C Fletcher

**Affiliations:** 1 Service de Psychiatrie, Centre Hospitalier Sainte-Anne, Université Paris Descartes, Sorbonne Paris Cité, Faculté de Médecine Paris Descartes, Paris, France; 2 Motivation, Brain, and Behavior Lab, Centre de Neuro-Imagerie de Recherche, Institut du Cerveau et de la Moelle épinière, Groupe Hospitalier Pitié-Salpêtrière, Paris, France; 3 INSERM U975, CNRS UMR 7225, UPMC-P6, UMR S 1127, Paris Cedex 13, France; 4 Department of Psychiatry and Behavioural and Clinical Neuroscience Institute, University of Cambridge, Cambridge, UK; 5 Laboratoire de "Physiopathologie des maladies Psychiatriques", Centre de Psychiatrie et Neurosciences U894, INSERM; Université Paris Descartes, Sorbonne Paris Cité, Paris, France; 6 Laboratoire de Neurosciences Cognitives (LNC), INSERM U960, Ecole Normale Supérieure (ENS), Paris, France; 7 Institute of Cognitive Neurosciences (ICN), University College London (UCL), London, UK; 8 Monash Clinical and Imaging Neuroscience, School of Psychological Sciences and Monash Biomedical Imaging, Monash University, Victoria, Australia; 9 Division of Anaesthesia, University of Cambridge, Cambridge, UK; 10 Addenbrooke‘s Hospital, Cambridge, UK; 11 Cambridge and Peterborough Foundation Trust, Cambridge, UK

## Abstract

A state of pathological uncertainty about environmental regularities might represent a key step in the pathway to psychotic illness. Early psychosis can be investigated in healthy volunteers under ketamine, an NMDA receptor antagonist. Here, we explored the effects of ketamine on contingency learning using a placebo-controlled, double-blind, crossover design. During functional magnetic resonance imaging, participants performed an instrumental learning task, in which cue-outcome contingencies were probabilistic and reversed between blocks. Bayesian model comparison indicated that in such an unstable environment, reinforcement learning parameters are downregulated depending on confidence level, an adaptive mechanism that was specifically disrupted by ketamine administration. Drug effects were underpinned by altered neural activity in a fronto-parietal network, which reflected the confidence-based shift to exploitation of learned contingencies. Our findings suggest that an early characteristic of psychosis lies in a persistent doubt that undermines the stabilization of behavioral policy resulting in a failure to exploit regularities in the environment.

## Introduction

One of the big challenges facing psychiatry is to develop an understanding of psychotic symptoms that goes beyond clinical description to uncover underlying computational and neurobiological mechanisms. A comprehensive account of the bizarre perceptions (hallucinations) and beliefs (delusions) that characterizes psychotic illness would require a mechanistic understanding of how the brain extracts and exploits regularities in the succession of events that occur in its environment. Reinforcement learning theory shows promise in this regard, by offering a framework within which we can consider causative disturbances at both the computational and neurobiological levels.^1, 2, 3^ Such perspectives might therefore give us the sort of mechanistic understanding that can ultimately shape diagnostic and therapeutic questions.

Insights derived from reinforcement learning models have already proven useful in developing theoretical accounts of how psychotic experiences may arise and how they may relate to disrupted brain processes. Previous empirical studies have focused on how prediction error signaling may be deranged in psychosis.^4, 5, 6, 7, 8^ Extending this several authors have suggested that the key deficit may reside not in prediction error *per se*, but rather in how prediction errors are used to update representations of the environment.^9, 10^ Of relevance, probabilistic learning tasks have been widely studied in schizophrenia (see refs. 11, 12, 13 for reviews), providing evidence for a complex pattern of deficit depending on the precise nature of the task (for example, complexity, occurrence and number of contingency reversals, explicit vs implicit learning) as well as of the profile of recruited patients (for example, predominantly positive vs negative symptoms, treated vs untreated patients). Interestingly, it has been proposed that the core impairment in schizophrenia might not affect learning ability *per se*, but rather the flexible control required to perform complex tasks and/or the capacity to optimize behavior in order to maintain a high level of performance.^11^ In line with such proposals, our hypothesis is that a key feature of early psychosis is a disruption in how confidence is updated and used to drive behavior in a dynamic environment.

In situations of low confidence (or elevated uncertainty), individuals may seek explanations, exploring various possibilities in an effort to identify regularities. Indeed, it has been demonstrated that in such situations, healthy subjects tend to perceive illusory patterns, creating regularities where there are none, and providing superstitious or conspiratorial explanations for ambiguous scenarios.^14^ These observations resemble the early features of psychosis, including sense of change and feeling of strangeness,^15, 16, 17^ search for explanation,^18, 19^ apophenia^20^ and jumping to conclusions.^21, 22^

Here, we sought to capture this transitory state in the context of an associative learning task implementing a dynamic environment. We predicted that, during learning of environmental contingencies, lack of confidence could lead to a reduced ability to stabilize an internal model of the world, with an ensuing, persistent sense of surprise. This would eventually result in sub-optimal behavior, characterized by an under-exploitation of true environmental regularities and an accompanying tendency to over-readily update in response to incidental violations of those regularities

Testing our predictions in a clinical setting is challenging given that, by the time psychosis is clearly identified, the expression of altered confidence may have been obfuscated by delusion formation and treatment effects. An established and fruitful solution is to use pharmacological models of early psychosis in healthy volunteers such as ketamine, a noncompetitive N-methyl-D-aspartate (NMDA) receptor antagonist^23, 24^ that induces subtle dissociative symptoms,^25^ perceptual learning alterations and, critically, psychosis-like experiences (see^26^ for a review). Here, we examined placebo-controlled, within-subject effects of a single dose of ketamine.

The task was adapted from previous paradigms.^27, 28^ On each trial, participants made a decision in response to a visual cue. The two options were always betting £1 versus betting 10p. The two options thus differed in risk, defined as the variance of possible outcomes. This does not imply that probability of winning was known, since it had to be learned by trial and error. This probability was 80% given one (positive) cue and 20% for the other (negative) cue. The optimal policy was to select the risky option following the positive cue and the safer option following the negative cue. To introduce instability into the environment, contingencies were reversed three times, such that the positive cue became the negative one and *vice-versa*. This task is close to tasks previously used to examine model learning under volatility (as in Behrens *et al.*^29^), except that transitions in probabilistic contingencies were not smooth but rather abrupt, as we wanted subjects to experience large variations in confidence, from the beginning to the end of learning blocks.

The key challenge posed to participants by our task was to notice unexpected outcomes that signaled a change in contingencies while ignoring those related to the probabilistic nature of these contingencies. Ignoring probabilistic errors requires confidence in the estimates of experimental regularities. Thus, we hypothesized that ketamine would prevent subjects from ignoring probabilistic errors, leading to sub-optimal behavior at the end of learning blocks, where subjects under placebo would fully exploit the learned contingencies. We explored the neural underpinnings of this ketamine-induced dysfunction, with the prediction that activity in confidence-related brain areas would show altered dynamics during the course of learning. Neural responses were concurrently tracked using functional magnetic resonance imaging (fMRI), while subjects performed the probabilistic contingency learning task. Each participant underwent this procedure during both ketamine and placebo infusions.

## Materials and methods

### Subjects

Twenty-one healthy, right-handed volunteers (11 males), aged 25–37 years (mean 28.7, s.d. 3.2), were recruited from the local community by advertisement, and screened using an initial telephone interview and subsequent personal interview. Exclusion criteria were: personal/familial history of neurological or psychiatric disorders, MRI contra-indications, illicit substance use in the last 12 months or any lifetime substance misuse syndrome or alcoholism, history of cardiac illness or high blood pressure, weight >10% above ideal body mass index. The study was approved by the Cambridge Local Research Ethics Committee, Cambridge, England, and was carried out in accordance with the Declaration of Helsinki. Written informed consent was given by all of the subjects.

### Ketamine infusion

Racemic ketamine (2 mg ml^−1^) was administered intravenously by initial bolus and subsequent continuous target-controlled infusion using a computerized pump (Graseby 3500; Graseby Medical, Watford, UK) to achieve plasma concentrations of 100 ng ml^−1^ using the pharmacokinetic parameters of a three-compartment model.^30^ One blood sample was drawn prior to the fMRI scan. Blood sample was placed on ice, plasma obtained by centrifugation and plasma samples stored at −70 ^o^C. Plasma ketamine concentration was measured by gas chromatography-mass spectrometry.

### Experimental design

**Figure 1:**
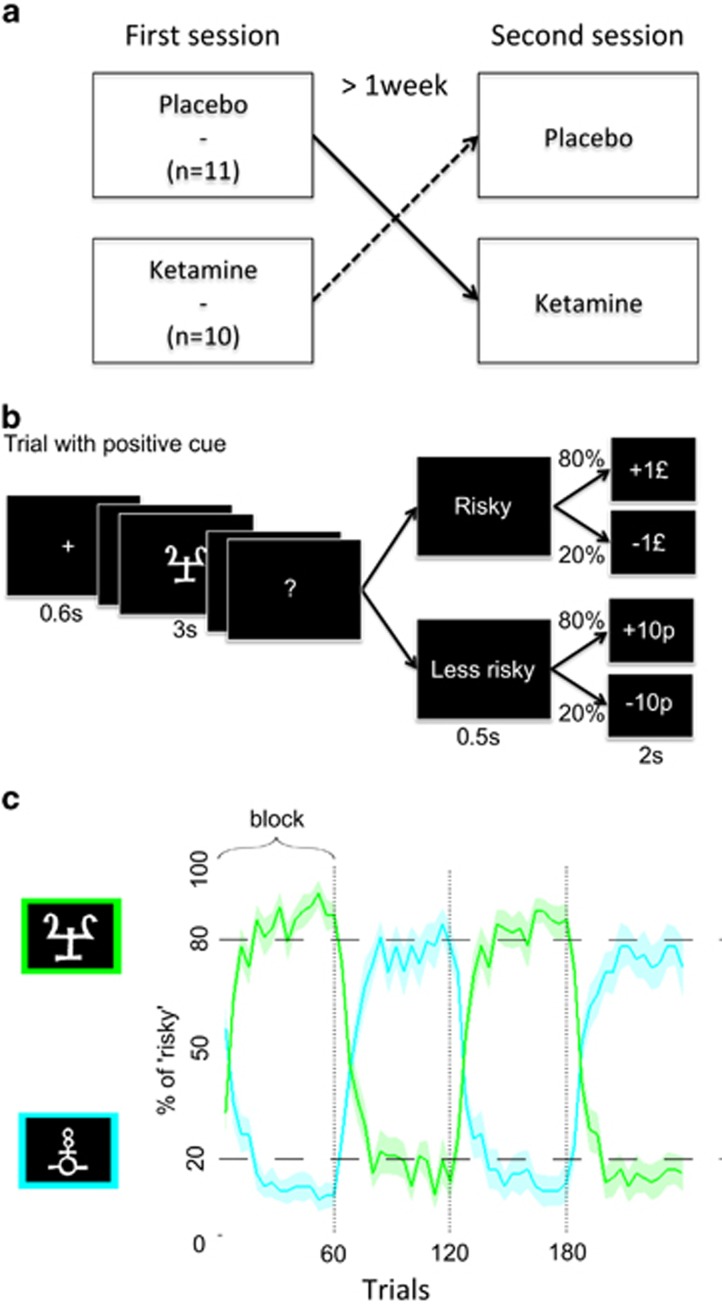
Experimental design. (**a**) A double-blind, placebo-controlled, randomized, within-subject design was used. The order of drug and placebo visits was counterbalanced across subjects and spaced by at least 1 week. (**b**) A typical trial and possible outcomes for a positive cue. Probabilistic contingencies (80 and 20%) would be swapped for a negative cue. (**c**) Percentage of risky responses as a function of trial number (both placebo and ketamine sessions were pooled). The green (respectively, blue) curve represents the choices following the cue that was positive (respectively, negative) in the first block. Bold lines represent means; color-delimited areas represent inter-subject s.e.m (corrected for the variance across subject: the grand mean of each subject was removed from its data before computing sem). Vertical dashed lines indicate reversals.

A double-blind, placebo-controlled, randomized, within-subjects design was used (see Figure 1a). At each visit, after starting the infusion of saline or low-dose ketamine, subjects underwent a clinical rating of positive psychotic symptoms as assessed by the Rating Scale for Psychotic Symptoms.^31^ Seven key items on the Brief Psychiatric Rating Scale^32^ representing symptoms of the psychosis prodrome (somatic concerns, anxiety-depression, elevated mood, grandiosity, hallucination and unusual thought content) were also assessed. Dissociative symptoms were assessed by the Clinician Administered Dissociative States Scale.^33^ Subjects then performed the probabilistic learning task in the fMRI scanner. Subjects also performed two other cognitive tasks while in the fMRI scanner. These were perceptual tasks not related to the current task and will not be reported here. Resting state data were also acquired.^34^

### Behavioral task

The task (see Figure 1) required participants, on each trial, to make a choice between a more and less risky option, indicating their choice by pressing a key or not. Risk taking was orthogonalized with respect to the motor dimension, so that pressing the key was assigned to the risky response only for half of participants and to the less risky response for the other half.

The risky (‘risk' being defined as the variance of the outcome) choice would lead to either the gain or the loss of £1, while the less risky option would lead to either the gain or loss of 10 pence. There were two contextual cues. One was associated with 80% chance of winning £1 (and a corresponding 20% chance of losing £1) following the risky choice and with 80% chance of winning 10 pence (and a 20% chance of losing 10 pence) following the less risky choice. For the other cue the contingencies were the opposite, that is, the risky choice would lead to an 80% chance of losing £1, while the less risky choice gave an 80% chance of losing 10 pence.

An unannounced contingency reversal occurred after each block of 60 trials (for a total of three reversals across the 240 trials). Reversal means that the positive cue (for which the risky choice was optimal) became the negative one and vice-versa. Therefore participants encountered the same contingency set only twice during the experiment.

Two abstract cues randomly taken among 24 letters from the Agathodaimon font were used. After fixation delay and cue display, the response interval was indicated on the computer screen by a question mark. The interval was fixed to 3 s and the response was taken at the end: this response was categorized as ‘risky'or ‘less risky' and was written on the screen as soon as the delay had elapsed. Monetary outcome was then displayed for 2 s. Participants were explicitly told that they would not receive the virtual money earned during the task. Instead, they were paid a fix amount that compensated for their time and their expenses associated with taking part in the study.

Before performing the task in the scanner, participants were familiarized with the task structure and with the notion that cue-outcome relationships were not necessarily constant. However, they were not warned that contingencies could be reversed.

### Model-free behavioral analysis

The overall percentage of risky response and button presses were compared between sessions in order to assess drug effects on choice and motor impulsivity, respectively. To assess drug effects on learning, the percentage of optimal responses (risky choice for the positive cue, less risky choice for the negative cue) were collapsed across the two cues and averaged within six bins of 10 consecutive trials. These data were then submitted to repeated-measure analysis of variance with three experimental factors (bin*block*session) and subjects as random factor. *Post-hoc* comparisons were performed to characterize the learning deficit observed under ketamine.

### Model-based behavioral analysis

The whole model space consisted of 27 models (see SOM): three variants of the reinforcement learning level without any confidence monitoring plus 24 variants of the hierarchical model (three reinforcement learning models × two ways to compute confidence × four ways to modulate low-level parameters) (see Figure 3 for a more detailed description of model space).

All models were inverted using a variational Bayes approach under the Laplace approximation,^35, 36, 37^ http://sites.google.com/site/jeandaunizeauswebsite/). This algorithm not only inverts nonlinear models but also estimates their evidence, which represents a trade-off between accuracy (goodness of fit) and complexity (degrees of freedom). The log-evidences estimated for each participant and model were submitted to a group-level random-effect analysis separately for placebo and ketamine sessions. To complete model selection, we also performed family analyses.^37^

### fMRI data analysis

fMRI data were preprocessed and statistically analyzed using SPM5 toolbox (Wellcome Department of Cognitive Neurology, London, UK) running on Matlab (Mathworks). T1-weighted structural images were coregistered with the mean functional image, segmented, and normalized to a standard T1 template and averaged across all subjects to allow group-level anatomical localization. The first five volumes of each session were discarded to allow for T1 equilibration effects. Preprocessing consisted of spatial realignment, normalization using the same transformation as structural images, and spatial smoothing using a Gaussian kernel with a full-width at half-maximum of 8 mm.

We devised two general linear models (GLM) to account for individual time series. The first GLM included separate categorical regressors for cue and outcome onsets, respectively, modulated by the computational variables, βm and αm. As parametric modulators were applied to different categorical regressors, they were not orthogonalized to each other. Note, however, that their correlation was quite low (*R*^2^=0.1) In the second GLM, outcome onsets were modulated by two computational variables, outcome category (confirmatory vs contradictory) and αm, that were serially orthogonalized, following on SPM default procedure. This second GLM was exclusively used for the region of interest (ROI) analysis. These variables were computed using subject-specific free parameters of the best fitting computational model (see computational results) and were then *z*-scored. All regressors of interest were convolved with a canonical hemodynamic response function. To correct for motion artifacts, subject-specific realignment parameters were modeled as covariates of no interest. Linear contrasts of regression coefficients were computed at the subject level and then taken to group-level random effect analyses.

Neural correlates of choice temperature and learning rate were identified in placebo sessions using a whole-brain one-sample *t*-test (cluster generating threshold *P*<0.001 uncorrected, cluster level threshold *P*<0.05 family-wise error corrected). The impact of ketamine on these networks was assessed using a paired *t*-test between ketamine and placebo sessions (cluster generating threshold *P*<0.01 uncorrected, cluster level threshold *P*<0.05 family-wise error corrected). In order to maximize sensitivity and to ensure that drug effects were only assessed within task-relevant networks, this analysis was masked by the parametric modulations (by choice temperature or learning rate) obtained when pooling placebo and ketamine sessions.

For ROI analyses, we extracted the regression estimates (betas) from spheres of 8mm in diameter (corresponding to the full-width at half-maximum of the Gaussian kernel used for spatial smoothing), centered on group-level activation peaks. The ventromedial prefrontal cortex (vmPFC) ROI, that was used to perform a comparison between placebo and ketamine session, was defined from the second-level analysis pooling both placebo and ketamine sessions in order to avoid biasing this comparison in favor of placebo sessions.

Additional GLMs were computed for illustrative purpose only. In these GLMs, trials were sorted in six bins of confidence (as defined in the best computational model) or trial number in a block (as in the model-free analysis: the first ten trials of each block, the following ten and so on). These GLMs were used to plot the hemodynamic response at cue and outcome onsets.

## Results

### Clinical assessments

The mean blood plasma concentration of ketamine during infusion was 96.01±19.11 ng ml^−1^. Paired *t*-tests indicated that ketamine caused a significant increase in positive psychotic symptoms as measured by the Rating Scale for Psychotic Symptoms (*t*(20)=5.43, *P*<0.001) and the Brief Psychiatric Rating Scale (*t*(20)=2.8, *P*=0.011), as well as in dissociative symptoms as measured by the Clinician Administered Dissociative States Scale (*t*(20)=3.72, *P*=0.0013).

### Behavioral results

**Figure 2:**
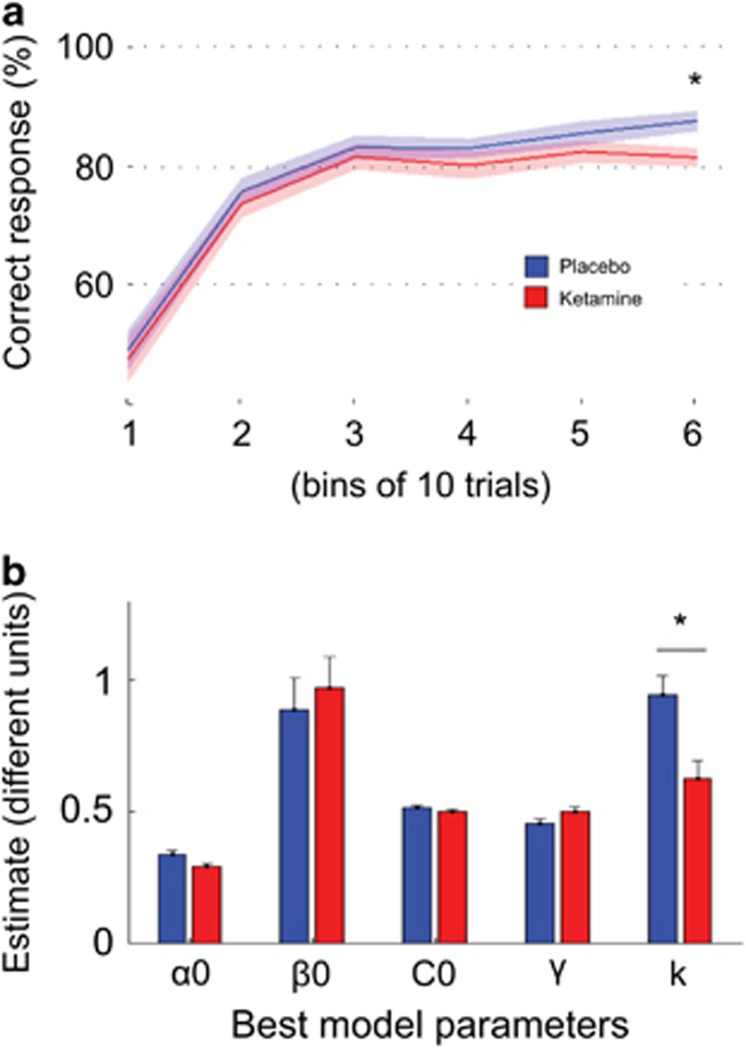
Characterization of the behavioral deficit induced by ketamine (**a**) Learning curves. Curves show percentage of correct response average across blocks, cues and bins of 10 consecutive trials, for the placebo (blue) and ketamine (red) sessions, separately. There was a significant effect of drug status in the last trial bin, with higher performance with placebo. Bold lines represent means; color-delimited areas represent inter-subject s.e.m (corrected for the variance across subject: the grand mean of each subject was removed from its data before computing s.e.m.). (**b)** Parameter estimates for the best computational model. The only parameter that significantly differed between sessions (placebo in blue versus ketamine in red) was *κ*, the weight that confidence had on learning rate and choice temperature. α0: learning rate value when confidence=0; β0: choice temperature value when confidence=0; C0: initial confidence value; *γ*: confidence learning rate. Bars represent means; error bars represent inter-subject s.e.m (corrected for the variance across subject: the grand mean of each subject was removed from its data before computing s.e.m.); *P<0.05, two-tailed paired *t*-test.

Choice and motor impulsivity did not differ between drug conditions (risky choice: 48.2% vs 47.8%, *t*(20)=0.29, *P*=0.8; button press: 53.0% vs 51.7%, *t*(20)=1.05, *P*=0.3). There was a main effect of learning, with optimal choices increasing across bins (F(5,100)=66.77, *P*<0.001), a main effect of block (F(3,60)=4.57, *P*<0.01) with more optimal choices during the first (pre-reversal) block (80%) compared with others (74%). There was no other main effect and no interaction between factors (all *P*>0.1). *Post-hoc* analysis showed a significant effect of drug status in the last trial bin (see Figure 2a), with higher performance under placebo (F(1,20)=5.641, *P*=0.028) without main effect nor interaction with block (both *P*>0.1). Indeed, during ketamine infusion, participants apportioned their responses in a way that matched or slightly exceeded the 80% probability of positive reinforcement (81.1%, *t*(20)=0.37, *P*=0.7 in comparison with 80%). In contrast, they optimized their behavior under placebo (87.2%, t(20)=2.52, *P*=0.02 compared to 80%). In summary, this preliminary behavioral analysis suggests that ketamine reduced the ability to go beyond probability matching, that is, to stabilize behavior in the face of probabilistic (misleading) unexpected outcomes. This hypothesis was formally assessed by using computational modeling.

### Computational modeling results

To explore a comprehensive set of possible strategies, we fitted qualitatively different models to the observed choices (see SOM for details). All models estimate the trial-wise values attached to the two cues, and use these values to predict choices, through a softmax function.

A first series of models were designed to account for low-level reinforcement learning. Following a standard ‘delta' rule,^38^ these models update after each trial the current cue value in proportion to prediction error, defined as the outcome value minus the expected value.

In a basic version, the outcome was simply the monetary amount (+£1, +0.1£, −0.1£ or -1£). In a second version, we integrated some understanding of the task structure by including the possibility that cue values were coded at a more abstract level, as if subjects figured out that all the information needed was the outcome valence (+ or −). In a third version the two cue values were updated after every outcome, to model the possibility that subjects realized that they always had an opposite valence, that is, information about the status of one cue also gave information about the status of the other.

Reinforcement learning models have constant parameters (learning rate α and choice stochasticity β). This limits the capacity to optimize the behavioral policy around the end of learning blocks, once subjects believe themselves to have a reasonably good estimation of contingencies. At this point, prediction errors should be tempered, and choices tuned to a more deterministic exploitation of learned contingencies.^29, 39, 40^ Conversely, when contingencies suddenly change after reversals, prediction errors should be given more weight, and choices should be more exploratory. This can be implemented in an optimal way using a hierarchical Bayesian architecture.^29, 40^ Some evidence has been found that human behavior can be accounted for by hierarchical Bayesian models.^41, 42^ However, Bayesian updates of probability distributions may become computationally cumbersome, and human subjects sometimes follow simpler heuristics, particularly when they are uncertain about the task structure.^43, 44^ Another way to optimize behavior is to subordinate the reinforcement learning parameters to a higher level of control that monitors performance. This idea has been proposed and formalized in the so-called meta-learning theoretical framework,^45^ which addresses the question of how machines can learn how to learn. This principle has been implemented for instance to adjust the exploration rate during the course of learning, and provides a good fit of nearly optimal primate behavior.^46, 47^

A second series of models followed this latter principle: they included a meta-cognitive level consisting in updating confidence (the belief that current representations are correct) so as to downregulate contingency learning and choice stochasticity. These hierarchical models allowed us to determine more precisely which level of learning was altered by ketamine infusion. Confidence was monitored using a delta rule in all the following models, which differed in the way outcomes were used to assess performance. A first variant used the absolute value of the prediction error generated in the lower reinforcement learning level, implementing the intuition that subjects should be more confident when prediction errors are reduced.^48, 49^ A second variant (following Khamassi *et al.*^47^) coded the outcome in terms of optimality: 0 for non-optimal outcomes (losing £1 or winning only 10p) and 1 for optimal outcomes (winning £1 or losing only 10p). In both variants, confidence could be used to modulate learning rate (αm), choice temperature (βm) or both, with different or identical weight. Optimizing choice temperature means favoring exploitation when confidence increases. Optimizing learning rate means increasing sensitivity to confirmatory outcomes and decreasing sensitivity to contradictory outcomes when confidence increases. Confirmatory means that the valence of the outcome is the same as the valence estimated by the model. Thus, when confidence was close to 0, the learning rate was similar for confirmatory and contradictory outcomes, but as confidence increased, it got closer to 1 for confirmatory outcomes and to 0 for contradictory outcomes.

**Figure 3:**
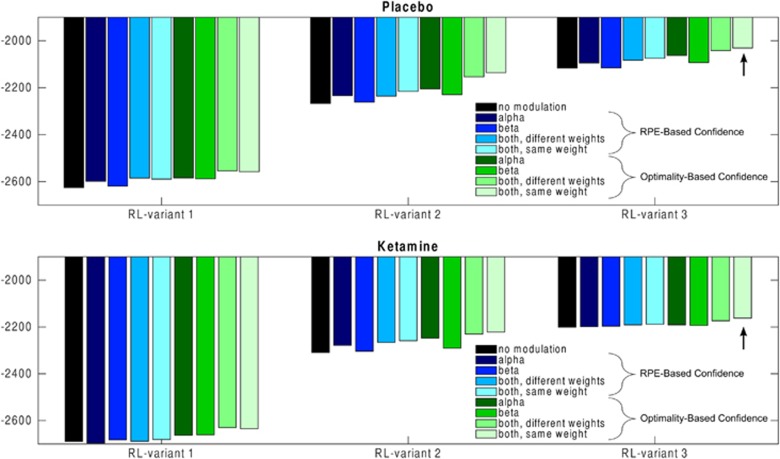
Model evidence (variational Bayesian approximation to marginal likelihood). The structure of the model space can be divided as follows. (i) Which RL variant? In variant 1, the reinforcer was the monetary amount; in variant 2, the reinforcer was the sign of the outcome; in variant 3, the reinforcer was the sign of the outcome and the two cue values were updated after every outcome. (ii) How to compute confidence? In a first variant, it was based on the absolute value of the prediction error. In a second variant, it was based on the optimality of the outcome, that is, 0 for non-optimal outcomes (losing £1 or winning only 10p) or 1 for optimal outcomes (winning £1 or losing only 10p). (iii) How to use confidence? Confidence was used to modulate the learning, choice temperature or both (with same or different weight). The arrow indicates the best model. Note that even the difference between the two rightmost bars is >10, which is considered to be a very strong difference in model evidence.

**Figure 4:**
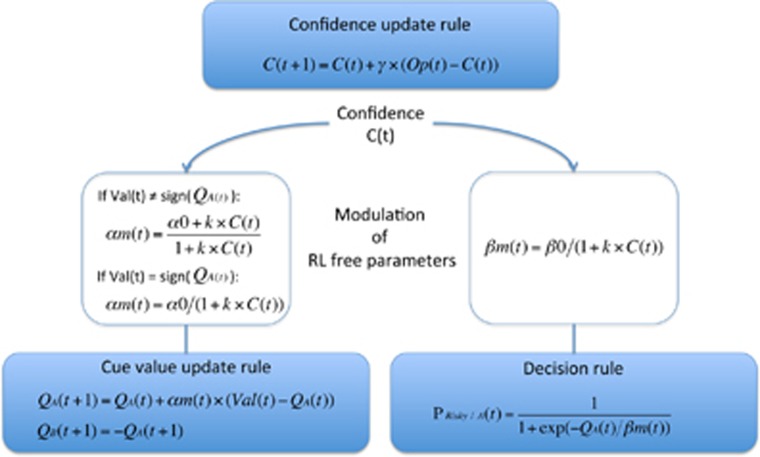
Description of the best model. The best model was selected using a group-level random-effect analysis. It included the third variant of RL (as if subjects figured out that only the outcome valence, and not the monetary amount, was informative about cue value, and that the two cues always had opposite valence such that they could both be updated after every outcome). Confidence was based on outcome optimality and used to modulate both the learning rate and choice temperature, with a same weight. Q is cue value; C is confidence; Op is outcome optimality (1 for winning £1 or losing 10p, -1 otherwise); Val is outcome valence (1 if positive, −1 otherwise); *P* risky/A is the probability of choosing the risky option when cue A is on screen. γ is confidence learning rate; α0 is learning rate value when confidence=0; β0 is choice temperature value when confidence=0; κ is the weight of confidence on learning rate and choice temperature.

Bayesian model selection was performed separately for placebo and ketamine sessions (see Figure 3 and Figure 4). The best model was the same in both sessions but the evidence was higher for placebo (xp=0.96; Supplementary Table S1) than for ketamine (xp=0.45; Supplementary Table S2). At the low level, this best model implemented an informed reinforcement learning rule, using the outcome valence (+ or −) to update the two cue values. At the high level, confidence was updated using the outcome optimality, and impacted both learning rate and choice temperature, with identical weights. Family model comparison^37^ confirmed that the best model was the same in both sessions though in ketamine sessions there was less clear evidence for the necessity of a meta-cognitive level that monitors confidence and allows confidence to modulate low-level parameters (see SOM for details).

We next compared the free parameters of this best model between placebo and ketamine sessions, with paired-tests (Figure 2b, Supplementary Table S3). The parameter that significantly differed between sessions was the weight that confidence had on learning rate and choice temperature (*t*(20)=2.3, *P*=0.027). Thus, ketamine reduced the impact of confidence on low-level parameters. This attenuation could therefore explain the deleterious effect of the drug on ability to optimize behavior when confidence increases, towards the end of learning blocks.

### Neuroimaging results

The computational analysis demonstrated that the behavioral effects of ketamine were underpinned by a shift in the dynamics of choice temperature and learning rate (βm and αm), which were insufficiently tuned by the confidence increases within learning blocks. To identify the underlying neural effects, we therefore focused on the neural representation of βm and αm, which, in principle, should be used to make choices at cue onsets and to update values at outcome onsets respectively. For each time point (cue and outcome onsets), we first analyzed the placebo session to identify the neural representation of βm or αm in the normal brain. We then directly compared placebo and ketamine sessions.

#### At choice onset

**Figure 5:**
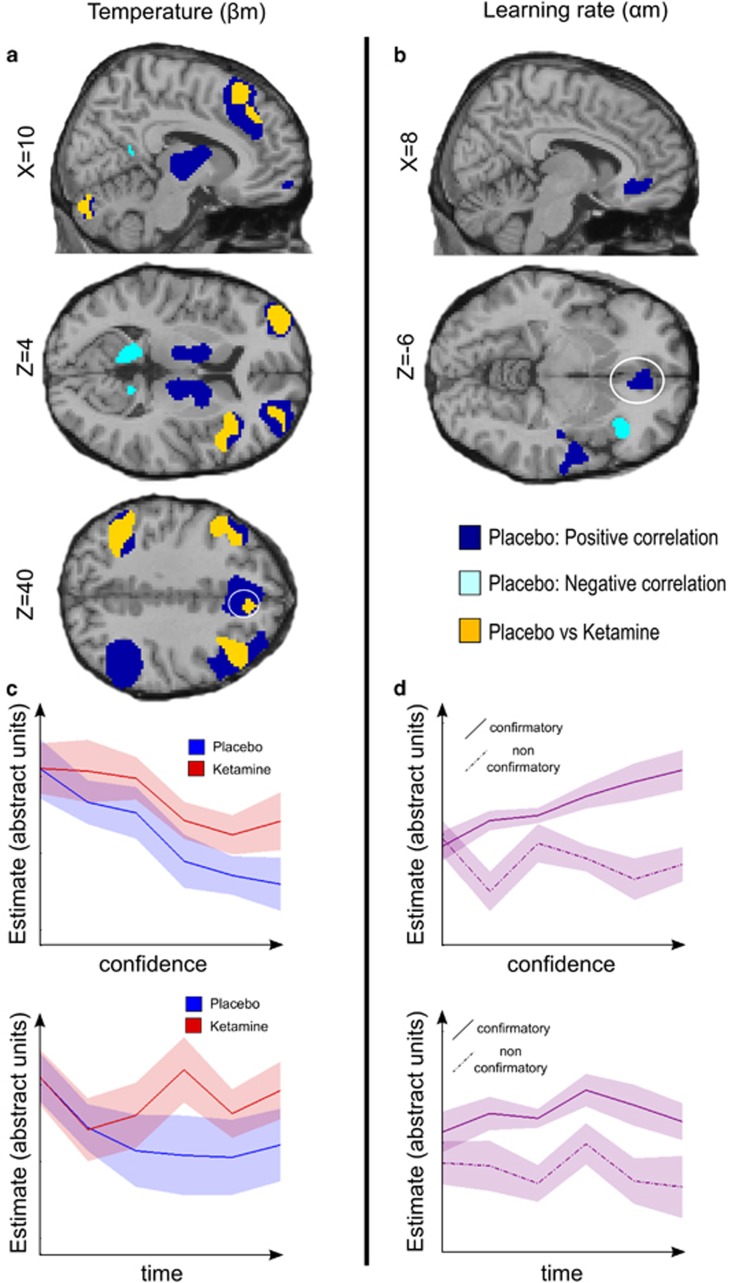
Model-based analysis of ketamine-induced changes in brain activity (**a**) Brain regions reflecting confidence-modulated choice temperature (βm). (**b**) Brain regions reflecting confidence-modulated learning rate (αm). Colored clusters show significant correlation in the placebo session (positive in dark blue, negative in light blue) and significant difference between placebo and ketamine sessions (in orange). All clusters survived a statistical threshold of *P*<0.05 after family wise error correction for multiple comparisons. Coordinates of anatomical slices are given in Montreal Neurological Institute space. (**c**) Hemodynamic response to cue onset in the dmPFC as a function of confidence bins or as a function of time (trial number, pooled across blocks) (for illustrative purpose). (**d**) Hemodynamic response to outcome onset in the ventromedial prefrontal cortex as a function of confidence bins or as a function of time (trial number, pooled across blocks) (for illustrative purpose), shown separately for confirmatory and contradictory outcomes. Placebo data are in blue, ketamine data are in red, pooled data are in violet. Bold lines represent means; color-delimited areas represent inter-subject s.e.m (corrected for the variance across subject: the grand mean of each subject was removed from its data before computing s.e.m.).

Under placebo, βm was correlated with activity in a large fronto-parietal network, including dorsomedial prefrontal cortex (dmPFC), frontopolar cortex and bilateral lateral prefrontal cortex. Other correlations were observed in the anterior insula, in addition to subcortical regions encompassing bilateral caudate nucleus, thalamus and cerebellum (Figure 5, Table 1). Put simply, elevated temperature was associated with enhanced activity in these regions. Conversely, βm was negatively correlated with activity in a bilateral network including cuneus, precuneus, posterior cingulate and medial temporal lobe.

In the ketamine session, the positive correlation with βm was significantly reduced compared to placebo in a bilateral fronto-parietal network, including the dmPFC, bilateral frontopolar cortex, bilateral lateral prefrontal cortex and left parietal cortex, as well as the anterior insula (Figure 5, Table 1). Thus, trial-to-trial variations in temperature expressed in the fronto-parietal network were diminished under ketamine. There was no significant difference between sessions for the negative correlation with βm.

#### At outcome onset

Under placebo, we observed a positive correlation with αm in the vmPFC and bilateral posterior insula extending to the superior temporal cortex (Figure 5, Table 2). These regions therefore increased their responses to confirmatory outcomes, and decreased their responses to contradictory outcomes, as confidence accumulated within learning blocks. Conversely, there was a negative correlation in the right anterior insula.

There was no significant difference in the correlation with αm between placebo and ketamine sessions at the whole-brain level, nor in a ROI analysis focusing on the vmPFC (*P*>0.1). Correlation with αm corresponds to an interaction between confidence and outcome category (confirmatory or contradictory). We verified that the correlation was not reducible to the main effect of outcome category: when this was regressed out, the correlation with αm was still significant in our vmPFC ROI under placebo (*t*(20)=2.79; *P*=0.01) but not under ketamine (*t*(20)=1.41; *P*=0.18), though the direct comparison was not significant (*P*>0.1). In short, under placebo but not ketamine, the difference between confirmatory and contradictory outcomes was amplified following the trial-wise increase in confidence within learning blocks.

## Discussion

Our working hypothesis was that early psychosis is characterized by a state in which the ability to acquire a robust and confident model of the world is lost. We tested this hypothesis at both the computational and neural levels, by combining a pharmacological model of early psychosis through NMDA blockade with model-based analysis of behavioral choices and fMRI data. The effects of NMDA blockade manifested in two ways:^1^ a decreased ability to optimize contingency learning in conditions of high confidence,^2^ a concurrent alteration in the regulation of brain systems reflecting choice stochasticity, notably in a bilateral fronto-parietal network including the dmPFC. Through use of a low dose of ketamine (rather than a higher one which would cause global cognitive difficulties), we have been able to identify a subtle and interpretable effect. Our findings have implications both for our understanding of contingency learning mechanisms and for theoretical perspectives on the emergence of psychosis. Because our experiment was carried out in a limited number of participants, as is common to pharmaco-MRI studies for obvious ethical reasons, we consider the implications below as primarily theoretical suggestions that will guide further investigations.

### Contingency learning mechanisms in an unstable environment

Our benchmark computational model was a standard Q-learning algorithm, which has been shown to provide a good account of instrumental learning in a variety of situations.^50^ However, the task was expressly designed such that Q-learning would not be optimal. This is because Q-learning gives a constant weight to outcomes in value updating, and a constant weight to value estimates in decision making. Yet it is adaptive to adjust these weights in unstable environments, where contingencies are stochastic and susceptible to sudden reversals, depending on the confidence in value estimates. Behavioral data suggested that participants did modulate choice and learning parameters as a function of confidence. To analyze this we developed a hierarchical model with a meta-cognitive level that monitors confidence and modulates first-level Q-learning parameters, an approach that has been formalized in the meta-learning framework.^45, 46, 51^ At the meta-cognitive level, Bayesian model selection indicated that an independent delta rule on outcome optimality (similar to that used in Khamassi *et al.*^47^) provided a better fit than a direct accumulation of unsigned prediction errors (as implemented in ^48^). Our construct of confidence can therefore be considered as surface monitoring, since it remains blind to the computations driving choices. In the model that best captured the behavioral data, both choice temperature and learning rate were dynamically adjusted as a function of confidence. Moreover, confidence had a differential impact on confirmatory outcomes (whose weight was amplified) and contradictory outcomes (whose weight was reduced). Together, these confidence-based adjustments enabled stabilizing internal representations of environmental contingencies (cue value estimates) and optimizing behavioral policy (exploitation of cue values).

Our concept of confidence can be linked to several recent theoretical propositions, in which higher level representations control lower-level processes. For example, it has been suggested that uncertainty, which quantifies ignorance about true values, drives the trade-off between exploitation and exploration.^52^ In the predictive coding framework, the precision of (or confidence in) beliefs determines the weight that prediction errors have in belief updating. Indeed, aberrant encoding of precision has been recently proposed to account for various aspects of psychosis.^10^ Some implementations of hierarchical Bayesian modeling can also be seen as very close to our approach, particularly when both the learning and decision rules are modulated by precision estimates.^42^ Note however that a new and important feature of our model is the differential impact of confidence on learning depending on the nature of the outcome (confirmatory or not), which allows neglect of contradictory information. We acknowledge that the concept of confidence is used for convenience, and corresponds in fact to a running estimate of performance. Whether this measure matches what participants would report as a feeling of confidence remains to be demonstrated.

Neuroimaging data provided additional support for our hierarchical model. At the time of cues, trial-wise variation in choice temperature was reflected in activation of a fronto-parietal network that has been previously implicated in cognitive control.^53, 54, 55, 56, 57^ This does not imply that all these regions have the function of representing choice temperature. Their activity might represent an indirect correlate of variations in this computational variable. In particular, regions such as the dmPFC has been involved in monitoring errors,^58, 59^ detecting conflicts^60, 61^ and making decisions under uncertainty.^59, 62, 63^ This region might signal the necessity of additional control, or even implement this necessary control, in periods of doubt regarding which choice is the best.^64, 65^ At the time of outcomes, trial-wise variation in learning rate was positively reflected in regions such as the vmPFC, which has been implicated in encoding the subjective value of stimuli.^66, 67^ Here, this region increased its response to confirmatory outcomes, and decreased its response to contradictory outcomes, from the beginning to the end of learning blocks. This finding extends a previous report that the vmPFC integrates option value and choice confidence^68^ by showing that this integration also applies to outcomes. Interestingly, the reverse pattern of activity was observed in the anterior insula, a region involved in signaling aversive values.^69, 70^ Thus, these two regions appeared to mediate the influence of meta-cognitive control on proximal reactions to gains and losses, such that they align to the distal goal of optimizing performance.

### Emergence of psychosis through NMDA blockade

Model-based analysis of the behavior suggested that NMDA blockade was associated with a reduced capacity to stabilize an internal model in order to capitalize on environmental regularities. This was evidenced by a reduced weight of confidence on choice temperature and learning rate. The performance deficit induced by ketamine infusion was therefore observed at the end of learning blocks, when confidence should be high enough to stabilize cue value estimation and exploitation policy. Our findings thus show that ketamine was associated with diminished ability to stabilize cue value estimates in the presence of probabilistic errors, as if a persistent doubt undermined optimization of behavior and made them more vulnerable to the effects of ‘noise' trials.

In a very simple environment as in our task (two cues with opposite values), such an impairment has limited impact and could hardly induce strange beliefs. In a more complex environment, where multiple internal explanatory models can be held at the same time, we would expect this impairment to forge strange beliefs, by combination of existing models or through the emergence of unexpected explanations. Our results therefore extend previous accounts of early psychosis, in which altered prediction errors lead to a sense of strangeness and to abnormalities in belief updating.^9, 15, 26^ Our findings suggest that it is important to take into account not just how prediction errors are used in low-level associative learning, but in how outcome optimality is integrated to modulate low-level parameters, via confidence monitoring.

We note that changes in key behavioral parameters did not correlate with the subtle psychopathology induced by this low dose of ketamine. This is perhaps unsurprising given the lack of statistical power—our experiment was devised with a view to identifying differences between ketamine and placebo rather than across-subject correlations. We see two other reasons that could account for this limitation. First, the neuro-cognitive perturbations that we demonstrated here might have different kinetics from those of psychotic symptoms (the former preceding the latter). Therefore, these two dimensions might remain uncorrelated at a given time. Second, if we assume that psychotic-like symptoms yield from more elementary cognitive dysfunctions, this link could be modulated (and hence blurred) by several factors, such as the existence of baseline (pre-ketamine) bizarre ideas, or the ability to introspect and conscious access to these dysfunctions and therefore to report psychotic-like symptoms.

In line with the behavioral analysis, the fMRI data showed that the confidence-based modulation of Q-learning parameters was significantly altered during ketamine infusion. Specifically, brain activity reflecting choice temperature was significantly less modulated by confidence under ketamine than placebo. This difference was observed in a bilateral fronto-parietal network, including the dmPFC. A detrimental effect of ketamine on dmPFC activation is in line with repeated observations of dorsal cingulate cortex impairment in patients with schizophrenia.^71^ Critically, here we offer a computational account of this effect, suggesting that that dmPFC impairment might play a key role in early symptoms of psychosis by compromising belief updating and policy adjustment in unstable environments. This dmPFC dysfunction could either alter confidence level or perturb the impact of confidence on behavioral policy.

The effect of ketamine on fronto-parietal regions might also relate to the well-established changes in consciousness produced by higher doses of ketamine,^72^ since the global workspace theory.^73, 74^ implicates these regions in conscious access by Interestingly, modulation of choice temperature by confidence was initially proposed to regulate the activity of workspace neurons whose role is to determine the degree of effort invested in decision making^46, 47^ in keeping with the concept of vigilance.^73^ One may speculate that the meta-cognitive component of our model, notably confidence monitoring and down-regulation of choice temperature, requires conscious processing. Thus, dysfunction of this part could be linked to both alteration of consciousness with higher doses of ketamine and to dysfunction of conscious processing in schizophrenic patients,^75, 76^ who would perform contingency learning in a more implicit way. Evidence for such a speculation would require further experiments manipulating consciousness levels.

The earliest stages of psychotic illness present an intriguing and puzzling set of cognitive changes. Computational psychiatry^1, 3^ offers new and rich frameworks for considering these changes and linking them to underlying neural alterations. Here we have shown that pharmacological fMRI, employing a well-established drug model of psychosis, presents a powerful tool in developing such frameworks, offering an opportunity to determine how controlled perturbations in glutamate function relate to altered balance in the dynamic control of optimal learning and behavior.

## Figures and Tables

**Table 1:** Brain regions reflecting confidence-modulated choice temperature (βm)

	*Structure*	*MNI coordinates (x, y, z)*	*Z-score*
Positive	Dorsomedial prefrontal cortex	−4, 24, 48	4.83
		48, 20, 52	4.78
	Dorsolateral prefrontal cortex	48, 28, 42	5
		36, 52, 24	4.31
		−28, 56, 26	4.18
		−46, 14, 44	4.26
		−38, 30, 42	4.24
		36, 54, −4	3.91
	Frontopolar cortex	14, 60, −10	4.66
		−42, 48, −4	4.05
		−22, 58, −6	4.49
		50, −52, 46	4.01
	(Inferior) parietal cortex	−42, −52, 42	4.73
		34, 22, −12	4.09
	Anterior insula	38, 22, 6	3.99
		−36, 18, −6	3.96
		40, −66, −50	3.74
	Cerebellum	−28, −74, −36	4.72
		16, 2, 14	4.6
	Caudate nucleus	10, −12, 0	4.17
	Thalamus	−8, −14, 0	4.35
Negative	Precuneus	−12, −48, 12	4.31
	Posterior cingulate	22, −52, 20	4.25
		−12, −58, 22	3.93
	Cuneus	22, −88, 26	3.41
	Medial temporal lobe	36, −38, −2	4.55
		−40, −36, −6	3.53
	Post-central gyrus	24, −48, 66	3.49
Placebo vs ketamine	Insula	48, 12, 8	3.77
		36, 20, 8	2.82
	Cerebellum	38, −66, −46	3.87
		−10, −82, −24	3.15
	Middle frontal gyrus	30, 18, 38	3.6
		−42, 16, 42	3.27
	Dorsomedial prefrontal cortex	6, 26, 60	3.07
		12, 26, 52	3.03
		−4, 26, 58	2.96
	Dorsolateral prefrontal cortex	40, 56, 20	2.81
		46, 30, 38	2.62
		−42, 16, 42	3.27
		−36, 12, 52	2.79
	(Inferior) parietal cortex	−54, −44, 42	3.26
	frontopolar cortex	−36, 54, 12	3.1
		42, 58, −4	2.67

**Table 2:** Brain regions reflecting confidence-modulated learning rate (αm)

	*Structure*	*MNI coordinates (x, y, z)*	*Z-score*
Positive	Posterior insula	58, −8, −2	4.52
		40, −18, −2	3.69
		−36, −18, −2	3.68
	Ventromedial prefrontal cortex	6, 36, −10	4.09
Negative	Anterior insula	38, 26, −6	3.7
