# Supplementary material for: Confidence and psychosis: a neuro-computational account of contingency learning disruption by NMDA blockade
Source: Mol Psychiatry. 2015 Jun 9;21(7):946–55. doi: 10.1038/mp.2015.73 (PMC5414075; doi:10.1038/mp.2015.73)
Supplement: Supplementary Information [file mp201573x1.pdf]

Supplemental Experimental Procedures

Description of model space

- Reinforcement learning level

We started with a basic, model-free reinforcement learning algorithm.(1) For each cue (say A or B), the model estimated the expected value, based on the individual outcome history, and made a choice between risky and less risky options. The expected values were set at zero before learning, and after each trial t the value of the ongoing cue (say A) was updated in proportion to prediction error, according to the 'delta' rule (2, 3):

$$Q_A\ (t+1) = Q_A\ (t) + \alpha \times \delta\ (t)$$

where δ(t) is the prediction error, defined as the difference between actual and expected outcome: $\delta(t) = R_Q(t) - Q_A(t)$.

Then the probability, or likelihood, of a "Risky" choice was estimated from the expected value according to the softmax rule:

$$P_{A\ Risky}\ (t) = 1/\ (1 + \exp^{(-Q_A(t)/\beta)})$$

The learning rate α and the choice temperature β are free parameters, with the constraints $0 \leq \alpha \leq 1$ and $\beta > 0$. The learning rate adjusts the weight assigned to prediction error in value updating, and the choice temperature the degree of exploration (as opposed to exploitation of the learned value).

We devised three variants of this reinforcement learning level, following step-by-step increments from model-free to model-based strategy, i.e. adding pieces of information about task structure.

In a first variant, the reinforcer R_Q was the monetary value of the outcome (1, 0.1, -0.1 or -1). This variant can be considered as a model-free strategy, in line with the law of effect, meaning that outcomes increased the probability of repeating the same choice, depending on their sign and magnitude.

In a second variant, the reinforcer R_Q was defined according to outcome valence (Val) and not its magnitude (i.e. 1 when winning £1 or 10p; -1 when loosing £1 or 10p). This variant implies that subjects understood that cues determined the outcome valence (positive or negative) and not its magnitude, which depended on the choice.

In a third variant, the reinforcer R_Q was defined according to outcome valence (Val) and the update of the current cue (say cue A) was transferred to the alternative cue (cue B).

Q_B (t+1) = -Q_A (t+1)

This variant implies that subjects understood that there were only two cues, with opposite valence. In other words, the two cue values summed up to zero.

- Meta-learning level

Reinforcement learning models have constant parameters (learning rate and choice stochasticity). This limits the capacity to optimize the behavioral policy around the end of learning blocks, once subjects believe themselves to have a reasonably good estimation of contingencies. At this point, prediction errors should be tempered, and choices tuned to a more deterministic exploitation of learned contingencies.(4-6) Conversely, when contingencies suddenly change after reversals, prediction errors should be given more weight, and choices should be more exploratory. One way to optimize the behavior is to subordinate the reinforcement learning parameters to a higher level of control that monitors performance. A second series of models therefore included a meta-cognitive level consisting in updating confidence so as to down-regulate contingency learning and choice stochasticity. We compared two ways to monitor confidence and four ways to use it.

- Confidence monitoring level

In both variants, confidence was monitored using a delta rule. The confidence learning rate γ was a free parameter, with $0 \leq \gamma \leq 1$. The initial value of confidence, C(0) was also fitted as a free parameter, with $0 \leq C(0) \leq 1$.

In a first variant, we used the absolute value of the prediction error computed at the reinforcement learning level to update confidence (7):

$$C(t+1) = C(t) + \gamma \times ((2-|\delta(t)|)/2 - C(t))$$

In a second variant, we used outcome optimality (Op) to update confidence (i.e. 1 for winning £1 or losing 10p, -1 otherwise):

$$C(t+1)= C(t) + \gamma \times (Op(t) - C(t))$$

- Modulation of low-level free parameters

Confidence was used to modulate the free parameters in the reinforcement learning models. This was done after each outcome, which brought information about how accurate the reinforcement learning model was, in terms of value estimates or behavioral policy. We considered four possibilities: modulation of learning rate or choice temperature, or both with the same weight, or both with a different weight.

The learning rate was modulated on the basis of not only confidence but also the outcome category. The idea is that to stabilize a representation of learned contingencies, subjects should increase their sensitivity to confirmation and decrease their sensitivity to contradiction. The impact of confidence on the learning rate α therefore depended on whether the outcome was confirmatory (outcome and cue value have the same sign; Val(t) = sign(Q(t))) or not.

For confirmatory outcomes, α was modulated as follows:

$\alpha m(t) = (\alpha 0 + k_\alpha * C(t))/(1+k_\alpha * C(t))$ where $\alpha 0$ and k_α are free parameter
And for contradictory outcomes:
$\alpha m(t) = \alpha 0/(1+ k_\alpha * C(t))$
Therefore, when confidence increased the modified learning rate αm got closer to 1 for confirmatory outcomes and closer to zero for contradictory outcomes.

The choice temperature β was modulated such that exploration was reduced when confidence increased:
$\beta m(t) = \beta 0/(1+k_\beta * C(t))$ where $\beta 0$ and k_β are free parameter
This modulation enables increasing exploitation above matching behavior, i.e. choosing the risky option more than 80% of the time following a cue that associated to a reward 80% of the time.
To test whether these modulations improved the fit of observed choices, we compared between models that included or not the free parameters (k_α and k_β), which could have or not identical values.

Other hierarchical models

Other hierarchical models have been developed to implement a form of second-level confidence that modulates first-level estimates. For instance, one hierarchical Bayesian architecture models the behavior in a probabilistic reversal learning task, with a second-level inference that tracks the occurrence of contingency reversals.(8) However, reversals were numerous in this task and participants were extensively trained, so they had built an internal model of the task (including the possibility of a reversal) before entering the scanner. In our paradigm, participants were not informed about the presence of reversals and they encountered only three of them, which was not enough to build and integrate an explicit notion of reversal at the meta-cognitive level. Unsurprisingly, we found no evidence that the behavior was more easily reversed the last time compared to the first one: if anything, performance in the last block was worst. For similar reasons, we did not include the possibility of re-using contingencies that were learned in previous blocks, as was done in another hierarchical model with task-set monitoring on top of Q-learning.(9) Indeed, there was no evidence that the second and third reversals, after which subjects could have returned to previous contingency sets, were learned faster than the first one. In addition, none of the existing models implemented the differential impact of the meta-cognitive level on the first-level learning rule, which enables participants to specifically ignore contradictory outcomes (probabilistic errors), a key way to stabilize behavior at the end of blocks. It is important to keep in mind that our paradigm was not designed to investigate reversal processes per se, but to examine how behavior is optimized between reversals.

Supplemental data

Family analyses

Family model comparison (10) was used to test whether each level of complexity added to the basic reinforcement learning model was necessary for explaining choice data. In a first comparison, the model space was divided into three families, depending on RL-variant (i.e. whether the monetary value or the outcome valence was integrated in the delta-rule and

whether the two cues or only the current cue was updated). Results confirmed that participants integrated the two aspects of task structure (xp =0.97 and 0.93 for placebo and ketamine sessions, respectively): first that only the outcome valence, and not monetary amount, was informative about cue value, and second that the two cues always had opposite valence such that they could both be updated after every outcome. In a second comparison, we divided the model space into three families, according to the way confidence was updated (see Fig 3: no confidence monitoring (and therefore no modulation), prediction error-based and optimality-based confidence updating. Evidence was higher for optimality-based confidence updating (xp=1 and 0.74 for placebo and ketamine sessions, respectively). In a third comparison, we divided the model space into five families, according to the way confidence was used (see Fig 3): no modulation (and therefore no confidence monitoring), modulation of learning rate, choice temperature, both with the same weight, or both with different weights. Results indicated that confidence was used to modulate both learning rate and choice temperature, with the same weight (xp=1 and 0.76 for placebo and ketamine sessions, respectively).

Correlation with psychotic-like symptoms

As our ultimate goal is to model delusion formation, we looked for correlations between psychotic-like symptoms induced by ketamine and our behavioural and imaging findings. For each of the 3 scales (RSPS, CADS, BPRS), the difference between placebo and ketamine sessions was computed for each subject and correlated to our behavioural markers of ketamine effect (decreased performance in the last bin and decreased weight of confidence on learning rate and choice temperature). No significant correlation was found. As far as imaging results are concerned, we also searched to correlate the increase of psychological symptoms to the reduction of the positive correlation with βm induced by ketamine. Scores were entered as a covariant in second-level GLM. No significant cluster was found.

Supplementary tables

Table S1
Exceedance probabilities table for Placebo sessions.

Table S2

Exceedance probabilities table for Ketamine sessions.

Table S3

Parameter estimates for the best computational model

Table S1

Placebo				
		RL - Variant		
Confidence – Monitoring Variant	Modulation of free parameters - Variant	1	2	3
0	0	0.00	0.00	0.00
1	1	0.00	0.00	0.00
1	2	0.00	0.00	0.00
1	3	0.00	0.00	0.00
1	4	0.00	0.00	0.00
2	1	0.00	0.00	0.00
2	2	0.00	0.00	0.00
2	3	0.00	0.00	0.00
2	4	0.00	0.02	0.96

Table S2

Ketamine				
		RL - Variant		
Confidence – Monitoring Variant	Modulation of free parameters – Variant	1	2	3
0	0	0.01	0.03	0.03
1	1	0.01	0.01	0.10
1	2	0.01	0.02	0.04
1	3	0.01	0.01	0.01
1	4	0.01	0.01	0.10
2	1	0.01	0.01	0.01
2	2	0.00	0.03	0.02
2	3	0.01	0.01	0.01
2	4	0.01	0.05	0.45

Table S3

	α0	β0	C0	γ	κ
Placebo	0.34 (0.12)	0.89 (0.74)	0.51 (0.04)	0.45 (0.15)	0.95 (0.39)
Ketamine	0.29 (0.16)	0.97 (1.17)	0.50 (0.04)	0.50 (0.16)	0.62 (0.53)

Supplementary references

1. Watkins CJ, Dayan P. Q-learning. Machine learning. 1992;8(3-4):279-92.
2. Rescorla RA, Wagner AR. A theory of Pavlovian conditioning: Variations in the effectiveness of reinforcement and nonreinforcement. Classical conditioning II: Current research and theory. 1972;2:64-99.
3. Sutton RS, Barto AG. Reinforcement learning, a Bradford book. MIT Press, Cambridge, MA; 1998.
4. Rushworth MF, Behrens TE. Choice, uncertainty and value in prefrontal and cingulate cortex. Nature neuroscience. 2008;11(4):389-97.
5. Behrens TE, Woolrich MW, Walton ME, Rushworth MF. Learning the value of information in an uncertain world. Nature neuroscience. 2007;10(9):1214-21.
6. Mathys C, Daunizeau J, Friston KJ, Stephan KE. A bayesian foundation for individual learning under uncertainty. Frontiers in human neuroscience. 2011;5:39.
7. Krugel LK, Biele G, Mohr PN, Li SC, Heekeren HR. Genetic variation in dopaminergic neuromodulation influences the ability to rapidly and flexibly adapt decisions. Proceedings of the National Academy of Sciences of the United States of America. 2009;106(42):17951-6.
8. Hampton AN, Bossaerts P, O'Doherty JP. The role of the ventromedial prefrontal cortex in abstract state-based inference during decision making in humans. The Journal of neuroscience : the official journal of the Society for Neuroscience. 2006;26(32):8360-7.
9. Collins A, Koechlin E. Reasoning, Learning, and Creativity: Frontal Lobe Function and Human Decision-Making. PLoS biology. 2012;10:e1001293.
10. Rigoux L, Stephan KE, Friston KJ, Daunizeau J. Bayesian model selection for group studies - revisited. NeuroImage. 2014;84:971-85.
